# Advanced lung cancer inflammation index predicts overall survival of hepatocellular carcinoma after hepatectomy

**DOI:** 10.3389/fonc.2024.1294253

**Published:** 2024-02-08

**Authors:** Yuan-Zhang Wen, Gao-Min Liu, Jia-Peng Liao, Ji-Wei Xu

**Affiliations:** ^1^ Department of Hepatobiliary Surgery, Meizhou Clinical Institute of Shantou University Medical College, Meizhou, China; ^2^ Department of Hepatobiliary Surgery, Meizhou People’s Hospital, Meizhou, China

**Keywords:** hepatocellular carcinoma, hepatectomy, advanced lung cancer inflammation index (ALI), survival, nomogram

## Abstract

**Aim:**

Limited data are available regarding ALI’s clinical relevance and prognostic value in patients with hepatocellular carcinoma (HCC) after hepatectomy.

**Materials and methods:**

HCC patients who received hepatectomy at the Meizhou People’s Hospital from May 2011 to February 2022 were enrolled in the study cohort. The ALI was calculated as follows: ALI = BMI (kg/m^2^) × ALB (g/dL)/(absolute neutrophil count/absolute lymphocyte count). The primary outcome was overall survival (OS). The secondary outcome was cancer-specific survival (CSS). Univariate and multivariate Cox regression analyses were performed, followed by nomogram construction and decision curve analysis (DCA).

**Results:**

425 HCC patients were enrolled for analyses. Lower preoperative ALI was significantly correlated with incomplete tumor capsule and advanced tumor stage. Lower preoperative ALI was an adverse independent prognostic factor for OS (HR: 1.512, 95% CI: 1.122-2.039, P 0.007) and CSS (HR: 1.754, 95% CI: 1.262-2.438, P <0.001) in HCC patients. The nomogram plot was built based on three (including age, TNM stage, and ALI) and two (including TNM stage and ALI) independent prognostic factors for OS and CSS, respectively. Further analyses indicated that the nomogram had better predictive value and some net benefit than the traditional TNM stage alone, especially in long-term OS.

**Conclusions:**

Our study further indicated that ALI could be a prognostic marker for OS and CSS in HCC patients after hepatectomy, especially in long-term OS.

## Introduction

Hepatocellular carcinoma (HCC) presents one of the most lethal global public health challenges (1). Upon diagnosis, the majority of patients lose the opportunity for curative treatments such as hepatectomy. Most patients experience recurrence and metastasis even after curative treatments within five years. Despite the significant advancements in surgical/systemic therapeutic approaches and multidisciplinary treatments, the overall prognosis of HCC is still far from satisfactory (2, 3). The importance of personalized management of HCC is increasingly emphasized (4). Therefore, it is crucial to investigate effective prognostic biomarkers to help identify high-risk HCC patients after hepatectomy.

Mounting evidence shows that malnutrition and persistent inflammation promote tumor occurrence, progression, recurrence, and metastasis (5). Traditional metabolic indicators (such as serum albumin (ALB), prealbumin (PA), and body mass index (BMI)) have been well implicated in various cancers, including HCC (6). In addition, markers reflecting systemic inflammation (such as neutrophil-lymphocyte ratio (NLR) (7) and lymphocyte-to-monocyte Ratio (LMR) (8)) can also serve as reliable indicators of liver cancer prognosis.

In 2013, a comprehensive indicator integrating ALB, BMI, and NLR was validated to predict survival of advanced non-small cell lung cancer (9), then this indicator was named the advanced lung cancer inflammation index (ALI). Subsequent studies suggest that ALI can effectively predict prognosis in various types of tumors (10–12). ALI was also found to predict HCC survival in patients with advanced HCC receiving immunotherapy (13, 14). However, the thorough prognostic value of the ALI for potentially curative HCC remained to be clarified.

In the present study, we investigated the prognostic values of the ALI in HCC patients who underwent surgical resection and identified high-risk patients to guide individual management after surgery. We present the following article following the STROBE reporting checklist.

## Materials and methods

### Study population

A total of 425 Han Chinese patients with HCC who were treatment naïve and underwent hepatectomy at the Meizhou People’s Hospital from May 2011 to February 2022 were included in the study cohort. The investigation, carried out at Meizhou People’s Hospital, received the endorsement of the Ethics and Indications Committee and it was executed in compliance with the guidelines outlined in the Declaration of Helsinki. The requirement for informed consent for this retrospective study was waived.

### Inclusion and exclusion criteria

The inclusion criteria were as follows: (I) all included patients were admitted to hospitals for primary diagnosis and were treatment-naïve; (II) patients were pathologically diagnosed with HCC; (III) patients received hepatectomy as the initial treatment; and (IV) all clinicopathological data for patients were available.

**Figure 1:**
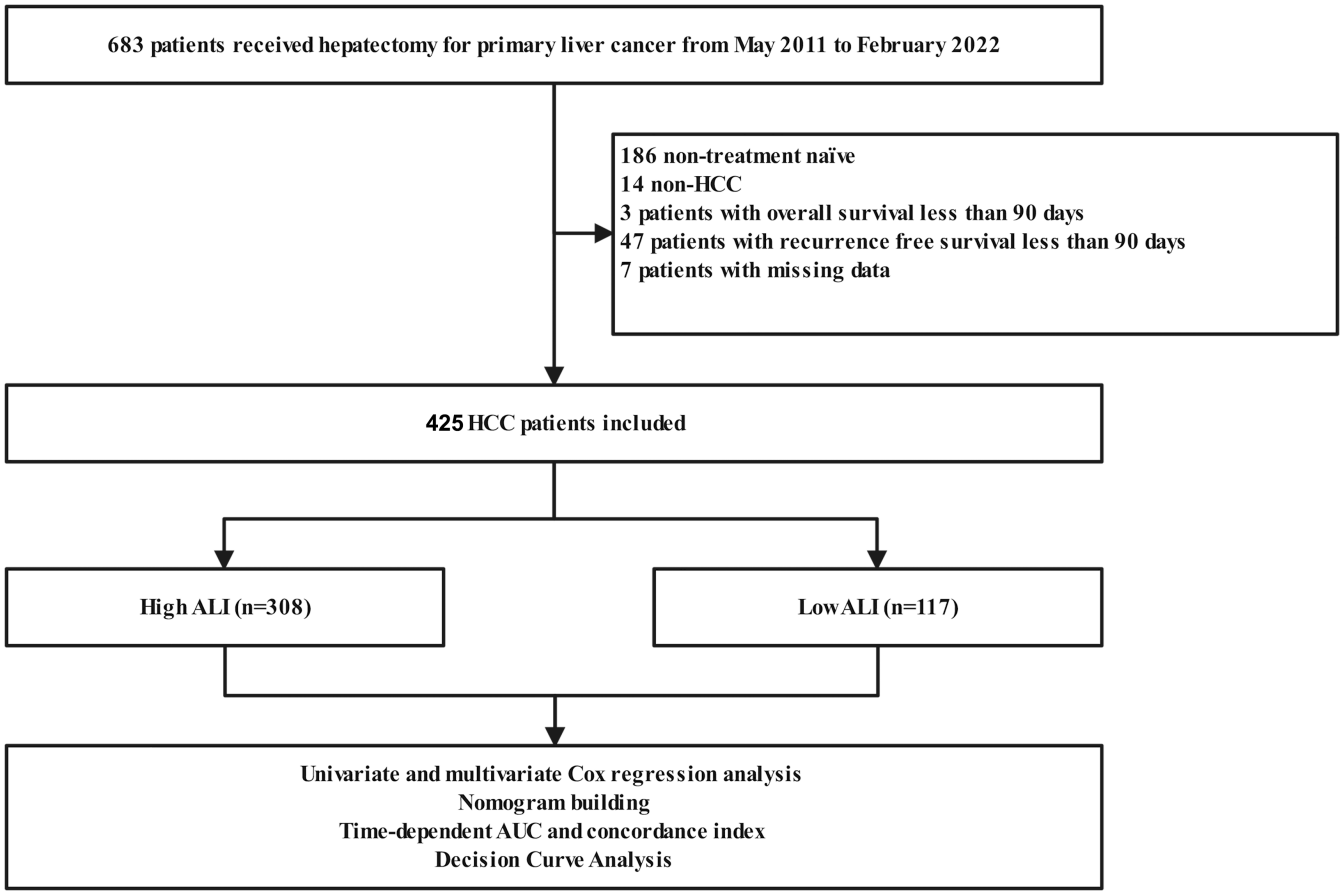
The flow chart of the study. ALI, advanced lung cancer inflammation index.

The exclusion criteria were as follows: (I) had an overall survival and recurrence-free survival of less than 90 days; (II) had non-HCC liver cancer; (III) had a history of malignant tumors other than primary liver cancer; (IV) received palliative resections. The process of patient selection was visualized in **Figure 1** through a flowchart.

### Data collection

All clinicopathological data were collected from the cancer database of the Meizhou People’s Hospital. The clinicopathological data included age, sex, weight, height, comorbidity (hypertension and diabetes), preoperative AFP, cirrhosis, tumor-specific data (number, grade, capsule, vascular invasion, microvascular invasion (MVI), and TNM stage), and survival data [overall survival (OS)]. Patients were classified according to the eighth edition of the International Union Against Cancer TNM classification system. The follow-up time is up to July 7th, 2023. BMI was calculated as weight divided by height squared. According to the literature, the ALI was calculated as follows: ALI = BMI (kg/m^2^) × ALB (g/dL)/(absolute neutrophil count/absolute lymphocyte count) (9). Furthermore, to account for the potential limitations in capturing all pertinent aspects of immune and metabolic status in HCC due to the specific weighting of the three parameters in the ALI formula, we conducted sensitivity analysis by adjusting the weights in the Cox regression model. The ALI was calculated in sensitivity analyses as follows: ALI = 0.0624*BMI (kg/m^2^) + 0.0307*ALB (g/dL) - 0.0135* (absolute neutrophil count/absolute lymphocyte count). The primary outcome was the OS. OS was defined as the period from the date of surgical resection of primary tumors to the date of death from any cause or until the last follow-up. The secondary outcome was the cancer-specific survival (CSS). CSS was defined as the period from the date of surgical resection of primary tumors to the date of death from cancer or until the last follow-up.

### Identification of independent prognostic factors and nomogram building

To identify independent prognostic factors, univariate and multivariate analyses were executed utilizing the Cox regression model with a forward stepwise procedure. The R package “survival” version 3.5-5 was used to conduct the analyses and plot the survival of the Kaplan–Meier survival curve. A nomogram (15) was constructed by incorporating every independent prognostic factor. To assess the calibration of the nomogram, the calibration plot was employed. The calibration was evaluated using a bootstrap technique with 1000 resampling iterations. TNM model, ALI, and the combined model including TNM and ALI were compared with time-dependent Area Under Curve (AUC), concordance-index, and the decision curve analysis (DCA) (16).

### Statistical analysis

Statistical analyses were performed using R software, version 4.3.1 (http://www.r-project.org). The R package “survminer” (version 0.4.9) was used to determine the optimal cut-off point of the ALI in this cohort. The qualitative variables were presented as the mean ± standard deviation or the median (interquartile range), meanwhile, the quantitative variables were shown as the count (percentage). Qualitative variables were compared with the Mann-Whitney U test or independent samples t-test, meanwhile, qualitative variables were assessed utilizing either the Pearson χ2 test or Fisher’s exact test. The survival analyses were plotted using the Kaplan–Meier method and were compared using the log-rank test. Two-sided P<0.05 was considered statistically significant.

## Results

### Baseline clinicopathological characteristics

A total of 425 patients were included in this analysis, including 380 men and 45 women, aged 28 to 85 years (median age, 58 years). In this cohort, 340 had stage I+II (80.00%), and 85 had stage III+IV (20.00%). The mean ALI score was 449.40 ± 386.67. The median follow-up for the 425 patients was 32.94 months (range, 3.02–129.40 months). 96 (22.86%) patients have died, with 74 (17.41%) of them succumbing to tumor recurrence and metastasis. The 1-, 3-, 5-, and 8-year OS rates in this cohort were 94.1%, 83.2%, 72.7%, and 52.0%, respectively. The 1-, 3-, 5-, and 8-year CSS rates in this cohort were 95.3%, 86.7%, 78.8%, and 64.9%, respectively.

### The correlation of ALI with clinicopathological characteristics

According to the R package “survminer”, the optimal cut-off value for the ALI for this cohort was 262.7. Subsequently, the 425 patients included in the study were categorized into high and low ALI groups, consisting of 308 and 117 patients, respectively. The correlation of ALI with clinicopathological parameters in HCC patients is summarized in **Table 1**. Lower preoperative ALI was significantly correlated with incomplete tumor capsule and advanced tumor stage (P=0.005 and P=0.001, respectively). However, some other well-defined adverse clinicopathologic factors were not significantly different between the two ALI groups, such as AFP, tumor number and grade, and vascular invasion.

**Table 1:** The correlation of ALI with clinicopathological parameters in HCC patients.

Characteristics	Level	Overall (n=425)	High ALI (n=308)	Low ALI (n=117)	P
ALI [mean (SD)]		449.40 (386.67)	557.01 (403.21)	166.10 (67.44)	<0.001
Age [mean (SD)] (Years)		58.16 (11.14)	58.12 (10.55)	58.40 (12.53)	0.816
Gender (%)	Male	380 (89.4)	272 (88.3)	108 (92.3)	0.308
	Female	45 (10.6)	36 (11.7)	9 (7.7)	
Hypertension (%)	No	344 (80.9)	242 (78.6)	102 (87.2)	0.060
	Yes	81 (19.1)	66 (21.4)	15 (12.8)	
Diabetes (%)	No	362 (85.2)	261 (84.7)	101 (86.3)	0.797
	Yes	63 (14.8)	47 (15.3)	16 (13.7)	
AFP (%) (ng/ml)	>400	128 (30.1)	94 (30.5)	34 (29.1)	0.861
	≤400	297 (69.9)	214 (69.5)	83 (70.9)	
Cirrhosis (%)	Without	169 (39.8)	117 (38.0)	52 (44.4)	0.270
	With	256 (60.2)	191 (62.0)	65 (55.6)	
Tumor number (%)	Single	341 (80.2)	244 (79.2)	97 (82.9)	0.474
	Multiple	84 (19.8)	64 (20.8)	20 (17.1)	
Tumor grade (%)	1	24 (5.8)	17 (5.6)	7 (6.1)	0.286
	2	280 (67.3)	198 (65.8)	82 (71.3)	
	3	106 (25.5)	83 (27.6)	23 (20.0)	
	4	6 (1.4)	3 (1.0)	3 (2.6)	
Tumor capsule (%)	Complete	371 (87.3)	278 (90.3)	93 (79.5)	0.005
	Incomplete	54 (12.7)	30 (9.7)	24 (20.5)	
Vascular invasion (%)	Without	330 (77.6)	237 (76.9)	93 (79.5)	0.667
	With	95 (22.4)	71 (23.1)	24 (20.5)	
MVI (%)	M0	245 (60.9)	182 (62.1)	63 (57.8)	0.679
	M1	89 (22.1)	64 (21.8)	25 (22.9)	
	M2	68 (16.9)	47 (16.0)	21 (19.3)	
TNM stage (%)	I+II	340 (80.0)	259 (84.1)	81 (69.2)	0.001
	III+IV	85 (20.0)	49 (15.9)	36 (30.8)	

AFP, alpha-fetoprotein; ALI, advanced lung cancer inflammation index; HCC, hepatocellular carcinoma; MVI, microvascular invasion; TNM, tumor-node-metastasis.

### Kaplan–Meier survival analysis

**Figure 2:**
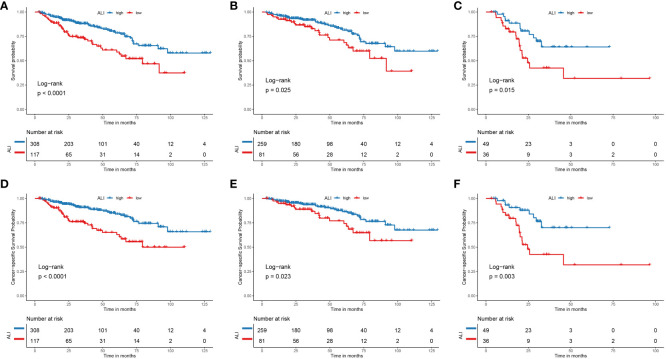
The Kaplan-Meier curve for the ALI in HCC. **(A)** The Kaplan-Meier curve showed the survival of patients was significantly poorer in patients with low ALI than in patients with high ALI. **(B)** The Kaplan-Meier curve showed the survival of patients was significantly poorer in stage I+II patients with low ALI than patients with high ALI. **(C)** The Kaplan-Meier curve showed the survival of patients was significantly poorer in stage III+IV patients with low ALI than patients with high ALI. **(D)** The Kaplan-Meier curve showed the cancer-specific survival of patients was significantly poorer in patients with low ALI than in patients with high ALI. **(E)** The Kaplan-Meier curve showed the cancer-specific survival of patients was significantly poorer in stage I+II patients with low ALI than patients with high ALI. **(F)** The Kaplan-Meier curve showed the cancer-specific survival of patients was significantly poorer in stage III+IV patients with low ALI than patients with high ALI. ALI, advanced lung cancer inflammation index; HCC, hepatocellular carcinoma; TNM, tumor-node-metastasis.

Kaplan-Meier survival analysis was performed to assess the prognostic influence of the ALI. For OS, patients with high ALI had a significantly better OS than those with low ALI [Hazard ratio (HR): 1.721, 95% confidence interval (CI): 1.288-2.299, P <0.001] (**Figure 2A**). Subsequently, patients were categorized based on their TNM stage. Consistently, patients with high ALI had a significantly better OS than those with low ALI, no matter in TNM stage I+II or TNM stage III+IV (P=0.025 and P=0.015, respectively) (**Figure 2B, C**).

For CSS, patients with high ALI had a significantly better CSS than those with low ALI [Hazard ratio (HR): 1.976, 95% confidence interval (CI): 1.430-2.730, P <0.001] (**Figure 2D**). Subsequently, patients were categorized based on their TNM stage. Similarly, patients with high ALI had a significantly better CSS than those with low ALI, no matter whether in TNM stage I+II or TNM stage III+IV (P=0.023 and P=0.003, respectively) (**Figure 2E, F**).

### Univariable and multivariable Cox regression analyses

**Figure 3:**
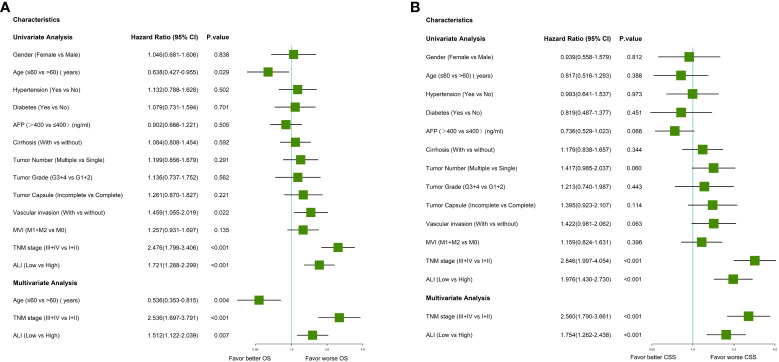
Forrest plot of the univariate and multivariate Cox regression analysis in HCC. **(A)** Forrest plot of the univariate and multivariate Cox regression analysis of overall survival; **(B)** Forrest plot of the univariate and multivariate Cox regression analysis of cancer-specific survival. AFP, alpha-fetoprotein; ALI, advanced lung cancer inflammation index; HCC, hepatocellular carcinoma; MVI, microvascular invasion; TNM, tumor-node-metastasis.

**Figure 4:**
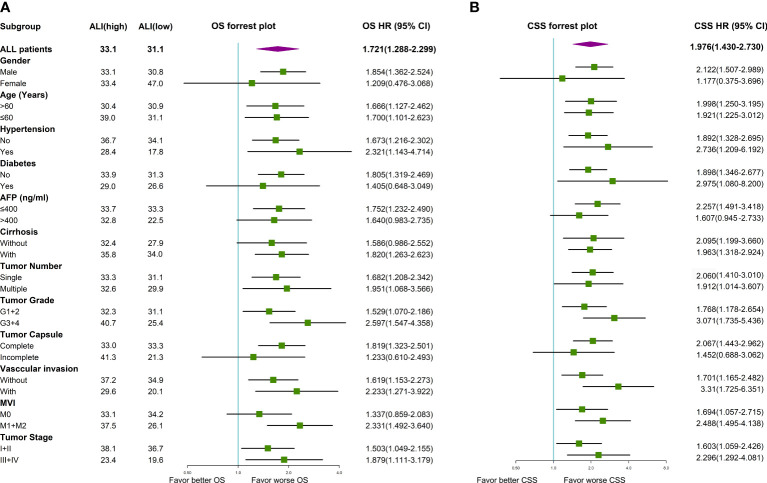
Forrest plot of the subgroup survival using univariate Cox regression analysis in HCC. **(A)** Forrest plot of the subgroup analyses for OS; **(B)** Forrest plot of the subgroup analyses for CSS. AFP, alpha-fetoprotein; ALI, advanced lung cancer inflammation index; CSS, cancer-specific survival; HCC, hepatocellular carcinoma; MVI, microvascular invasion; OS, overall survival; TNM, tumor-node-metastasis.

Univariate Cox regression analysis indicated that older age, vascular invasion, advanced TNM stage, and low ALI were associated with worse OS in this cohort. Then these four prognostic factors were included in the multivariable Cox regression analyses. As shown in **Figure 3**, older age, advanced TNM stage (HR: 2.536, 95% CI: 1.697-3.791, P <0.001), and low ALI (HR: 1.512, 95% CI: 1.122-2.039, P=0.007) were both independent prognostic factors for OS. Then subgroup survival analyses were conducted. As shown in **Figure 4A**, low ALI was significantly correlated with worse OS in most subgroups.

Univariate Cox regression analysis indicated that multiple tumors, advanced TNM stage, and low ALI were associated with worse CSS in this cohort. As shown in **Figure 3**, the advanced TNM stage (HR: 2.560, 95% CI: 1.790-3.661, P <0.001) and low ALI (HR: 1.754, 95% CI: 1.262-2.438, P <0.001) were both independent prognostic factors for CSS. Then subgroup survival analyses were conducted. As shown in **Figure 4B**, low ALI was significantly correlated with worse CSS in most subgroups.

### Sensitivity analysis

According to the R package “survminer”, the optimal cut-off value for the ALI in the sensitivity analysis for this cohort was 2.533. For OS, patients with high ALI had a significantly better OS than those with low ALI (Hazard ratio (HR): 1.640, 95% confidence interval (CI): 1.231-2.185, P <0.001) (**Supplementary Figure 1A**). Subsequently, patients were categorized based on their TNM stage. However, patients with high ALI had a significantly better OS than those with low ALI only in TNM stage I+II but not in TNM stage III+IV (P<0.001 and P=0.640, respectively) (**Supplementary Figure 1B, C**). For CSS, patients with high ALI had a significantly better CSS than those with low ALI (Hazard ratio (HR): 1.755, 95% confidence interval (CI): 1.270-2.426, P <0.001) (**Supplementary Figure 1D**). Subsequently, patients were categorized based on their TNM stage. Similarly, patients with high ALI had a significantly better CSS than those with low ALI only in TNM stage I+II but not in TNM stage III+IV (P<0.001 and P=0.340, respectively) (**Supplementary Figure 1E, F**).

As shown in **Supplementary Figure 2**, older age, advanced TNM stage (HR: 2.697, 95% CI: 1.831-3.971, P <0.001), and low ALI (HR: 1.541, 95% CI: 1.154-2.058, P=0.003) were both independent prognostic factors for OS. Subgroup survival analyses also found that low ALI was significantly correlated with worse OS in most subgroups (**Supplementary Figure 3A**). As shown in **Supplementary Figure 2**, advanced TNM stage (HR: 2.671, 95% CI: 1.875-3.805, P <0.001), and low ALI (HR: 1.624, 95% CI: 1.172-2.250, P=0.004) were both independent prognostic factors for CSS. Subgroup survival analyses also found that low ALI was significantly correlated with worse CSS in most subgroups (**Supplementary Figure 3A**).

Taken together, these sensitivity analyses further validated the robustness of ALI’s prognostic role in HCC after hepatectomy.

### Nomogram building and DCA curve

**Figure 5:**
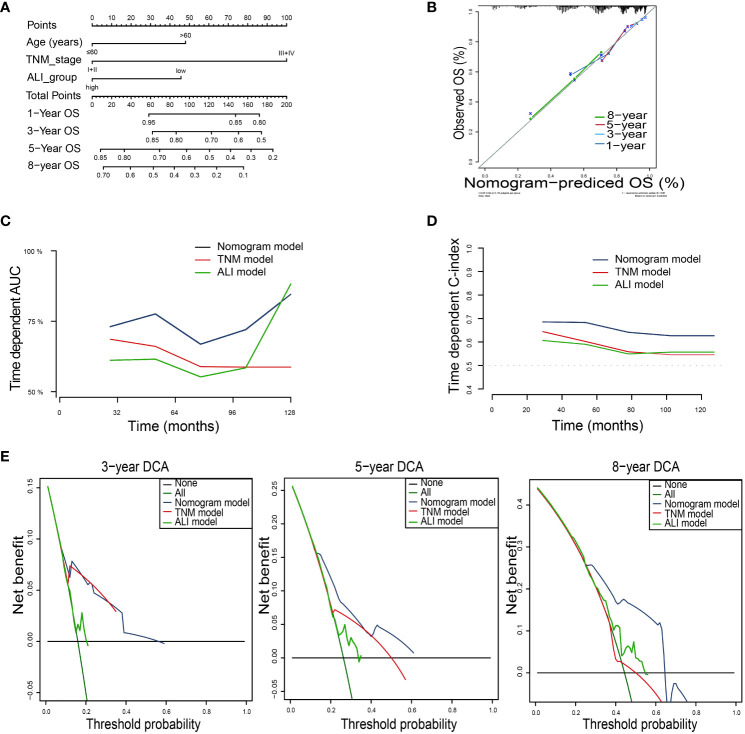
Building the nomogram predicting overall survival for HCC patients. **(A)**The nomogram plot was built based on three independent prognostic factors in HCC. **(B)** The calibration plot for internal validation of the nomogram. **(C)** The time-dependent AUC curves compare the nomogram, TNM model, and ALI model, respectively. **(D)** The time-dependent concordance index curves compare the nomogram, TNM model, and ALI model, respectively. **(E)** The DCA curves of the nomograms compared for 3-, 5-, and 8-year overall survival in HCC, respectively. HCC, hepatocellular carcinoma; TNM, tumor-node-metastasis.

For OS, the nomogram was built by including age, TNM stage, and the ALI (**Figure 5A**). Calibration plots showed that the performance of the nomogram was best in predicting 3-year OS (**Figure 5B**). The C-index was 0.63, 0.60, and 0.68 for the TNM, ALI, and nomogram models, respectively. The AUC of the nomogram for 1-year, 3-year, 5-year, and 8-year OS were 0.64, 0.75, 0.75, and 0.71, respectively. Meanwhile, the AUC of the TNM model for 1-year, 3-year, 5-year, and 8-year OS were 0.61, 0.71, 0.65, and 0.57, respectively. The AUC of the ALI for 1-year, 3-year, 5-year, and 8-year OS were 0.61, 0.60, 0.56, and 0.61, respectively. The superiority of the nomogram over the TNM or ALI model alone was further demonstrated by the time-dependent AUC and C-index curve (**Figures 5C, D**). Combining the ALI with the TNM stage showed some net benefit for predicting OS, especially for 5-year and 8-year survival (**Figure 5E**).

**Figure 6:**
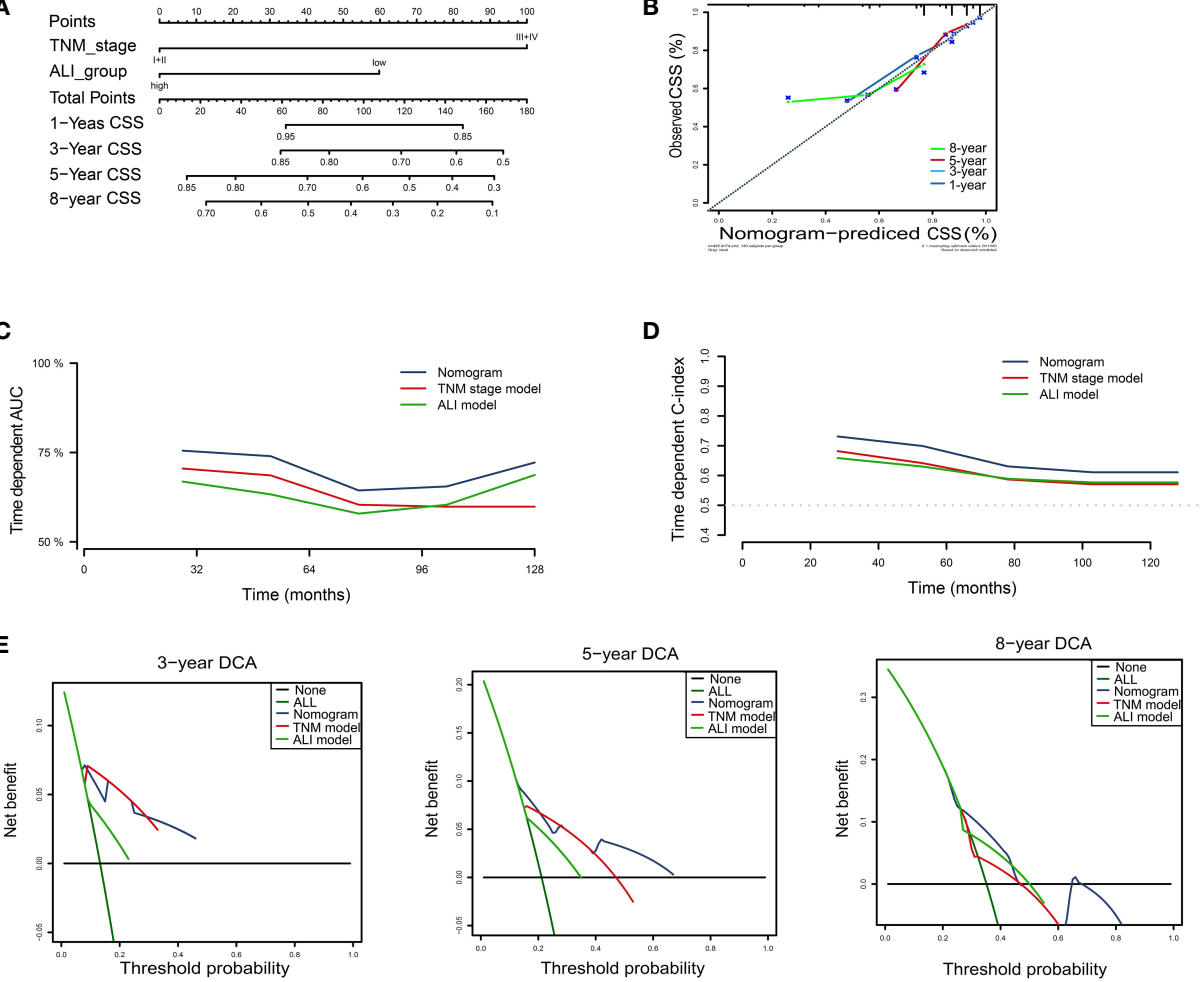
Building the nomogram predicting cancer-specific survival for HCC patients. **(A)**The nomogram plot was built based on three independent prognostic factors in HCC. **(B)** The calibration plot for internal validation of the nomogram. **(C)** The time-dependent AUC curves compare the nomogram, TNM model, and ALI model, respectively. **(D)** The time-dependent concordance index curves compare the nomogram, TNM model, and ALI model, respectively. **(E)** The DCA curves of the nomograms compared for 3-, 5-, and 8-year cancer-specific survival in HCC, respectively. CSS, cancer-specific survival; HCC, hepatocellular carcinoma; TNM, tumor-node-metastasis.

For CSS, the nomogram was built by including the TNM stage and the ALI (**Figure 6A**). Calibration plots showed that the performance of the nomogram was best in predicting 3-year CSS (**Figure 6B**). The C-index was 0.65, 0.63, and 0.70 for the TNM, ALI, and nomogram models, respectively. The AUC of the nomogram for 1-year, 3-year, 5-year, and 8-year CSS were 0.70, 0.76, 0.69, and 0.66, respectively. Meanwhile, the AUC of the TNM model for 1-year, 3-year, 5-year, and 8-year OS were 0.65, 0.73, 0.68, and 0.58, respectively. The AUC of the ALI for 1-year, 3-year, 5-year, and 8-year OS were 0.63, 0.62, 0.59, and 0.62, respectively. The superiority of the nomogram over the TNM or ALI model alone was also further demonstrated by the time-dependent AUC and C-index curve (**Figures 6C, D**). Combining the ALI with the TNM stage showed some net benefit for predicting CSS, especially for 5-year and 8-year CSS at certain thresholds (**Figure 6E**).

## Discussion

In this study, we investigated the associations between ALI and clinical characteristics and explored the prognostic significance of ALI in HCC patients who underwent potential curative hepatectomy. We found that lower preoperative ALI was significantly correlated with incomplete tumor capsule and advanced tumor stage. We also found that low ALI was an adverse independent prognostic factor for OS and CSS in HCC patients. Sensitivity analyses further prove the robustness of ALI’s prognostic role in HCC after hepatectomy and combining the ALI with the TNM stage showed some net benefit for predicting OS and CSS.

Systemic inflammation response not only contributes to the development and progression of cancer but also contributes to the treatment response of cancer (17). Currently, systemic inflammation is emerging as a prominent hallmark of cancer (18). From this point of view, it is reasonable that biomarkers indicating systemic inflammation might play a significant role in both the diagnosis and prognosis of cancer. Having the capacity to evaluate both systemic inflammation and metabolic status comprehensively, the prognostic significance of ALI has been confirmed across diverse cancer types, including HCC (19). Systemic inflammation also could provide useful guidance for cancer treatment with immunotherapies (20, 21). Recently, Li et al. also reported that ALI could be used as a novel prognostic marker in patients with advanced HCC receiving immunotherapy (13, 14). Nevertheless, previous ALI studies mostly focused on other cancers such as lung cancer. Limited data are available regarding ALI’s clinical significance and prognostic value in patients with hepatocellular carcinoma (HCC) who undergo initial hepatectomy. Our study offered significant evidence indicating that, beyond the TNM stage, ALI could offer crucial prognostic value in this patient population.

Interestingly, while the combination of ALI with the TNM stage demonstrated a net advantage in predicting OS and CSS in HCC, the most noticeable net benefit emerged five years post hepatectomy. As the follow-up period progressed, the predictive ability of TNM for survival diminished, while the predictive capacity of ALI became stronger. As is commonly understood, the leading cause of mortality in HCC is cancer recurrence or metastasis. Early recurrence was primarily influenced by factors related to the initial tumor, such as vascular invasion and TNM stage. In contrast, late recurrence was associated with factors linked to the underlying hepatitis condition, contributing to the development of *de novo* HCC (22, 23). From this perspective, our discovery could potentially indicate the distinct mechanisms influencing the short-term and long-term survival of HCC. ALI, functioning as an indicator of systemic inflammation and metabolic status of the underlying liver condition, might exert a stronger influence on late recurrence, thereby impacting long-term survival. The utilization of ALI could be particularly beneficial in the extended management of HCC.

An increasing number of second-line treatment options such as anti-angiogenic agents and immunotherapy have shown promising therapeutic efficacy in HCC (24). Systemic inflammation, as reflected by ALI to some degree, is increasingly recognized as a hallmark of cancer, including HCC. ALI before immunotherapy is a strong predictor for disease control and provides great predictive value for metastatic melanoma patients treated with immunotherapy as second-line therapy (25). In HCC, ALI was also found to predict HCC survival in patients with advanced HCC receiving immunotherapy (13, 14). Nevertheless, the link between ALI, systemic inflammation, and treatment response remains to be elucidated. Besides, anti-angiogenic agents are more widely used as second-line treatments for HCC. The presence of vascular invasion is a crucial determinant of prognosis and response to anti-angiogenic therapies. In this study, ALI was not significantly correlated with vascular invasion or MVI. However, the potential role of ALI as a predictive marker for response to anti-angiogenic treatments warrants further investigations. In addition, some patients also received anti-angiogenic agents and/or immunotherapy after HCC recurrence. The extent to which these treatment histories influence the predictive capacity of ALI and the established nomogram for HCC survival remains uncertain. Future research should aim to elucidate the role of ALI or its dynamic changes in patients receiving second-line treatments, or in combination with second-line treatment history beyond hepatectomy for HCC prospectively. Specifically, there is a necessity for validation studies targeted specifically at patients who have undergone second-line treatments. Although we demonstrated the clinical usefulness of ALI in HCC patients who received initial hepatectomy, several limitations need to be pointed out. First, the study design of the study could introduce inherent biases. For instance, being a single-center study and not including non-Asian populations will restrict the generalizability of our findings. The retrospective study design may introduce inherent selection bias and pose challenges in controlling confounding factors. The overly stringent exclusion criteria, particularly the requirement for a minimum overall survival and recurrence-free survival of 90 days, might lead to selection bias, limiting the interpretation of ALI’s value on short-term survival. Nevertheless, we maintain these exclusion criteria to minimize potential interference from perioperative complications, residual tumors post-resection, and non-standardized follow-ups in interpreting the outcomes. Additionally, the follow-up time may be relatively short for assessing long-term outcomes, as a considerable portion of patients have not yet experienced death or recurrence. Second, some issues remain ambiguous in our study and warrant further demonstrations, such as the correlation of ALI with some important clinicopathological parameters (such as AFP and MVI) and the clear prognostic role of ALI in TNM stage III+IV. Third, the established nomogram lacked external validation to further test its predictive value. Further prospective multicenter studies with larger sample sizes are urgently needed. These studies should encompass longer follow-ups, expanded variable inclusion (such as treatment modalities, genetic factors, and lifestyle aspects), and different racial populations to deepen our understanding of ALI’s clinical relevance and enhance the robustness and validity of our findings. Without such validation, the utilization of ALI as a prognostic marker is characterized more by optimistic speculation than by a definitive claim. Fourth, several aspects were not covered in this study. These include exploring the clinical significance of the dynamic changes in ALI during the treatment process, unraveling the underlying mechanisms connecting ALI to HCC survival, investigating the influence of ALI on patient-reported outcomes, examining its prognostic relevance in patients undergoing diverse treatment modalities beyond hepatectomy, and assessing the implications of integrating novel technologies such as omics or artificial intelligence. In addition, some other well-known biomarkers of inflammation, such as erythrocyte sedimentation rate (ESR), C-reactive protein (CRP), and plasma viscosity (PV), were not included in the analysis due to lack of data. Integrating these data for analysis will be even more helpful in elucidating the prognostic value of ALI for HCC after hepatectomy.

## Conclusions

Our study demonstrated that lower preoperative ALI was significantly correlated with incomplete tumor capsule and advanced tumor stage and indicated adverse independent OS and CSS in HCC patients who underwent potential curative hepatectomy. Combining the ALI with the TNM stage showed some net benefit for predicting OS and CSS, especially in the long-term OS. Nevertheless, further validation through larger prospective multi-center studies is necessary.

## Data availability statement

The original contributions presented in the study are included in the article/**Supplementary Material**. Further inquiries can be directed to the corresponding author.

## Ethics statement

The studies involving humans were approved by Meizhou People’s Hospital (No. 2019-77). The studies were conducted in accordance with the local legislation and institutional requirements. The ethics committee/institutional review board waived the requirement of written informed consent for participation from the participants or the participants’ legal guardians/next of kin because the requirement for informed consent was waived for the retrospective study.

## Author contributions

Y-ZW: Data curation, Formal analysis, Writing – original draft. J-PL: Data curation, Software, Visualization, Writing – original draft. J-WX: Conceptualization, Investigation, Supervision, Visualization, Writing – review & editing. G-ML: Conceptualization, Validation, Visualization, Writing – review & editing, Data curation, Formal analysis, Funding acquisition.

## Supplementary material

Supplementary Figure 1 The Kaplan-Meier curve for the ALI in HCC. **(A)** The Kaplan-Meier curve showed the survival of patients was significantly poorer in patients with low ALI than in patients with high ALI. **(B)** The Kaplan-Meier curve showed the survival of patients was significantly poorer in stage I+II patients with low ALI than patients with high ALI. **(C)** The Kaplan-Meier curve showed the survival of patients was not significantly different in stage III+IV patients with low ALI than patients with high ALI. **(D)** The Kaplan-Meier curve showed the cancer-specific survival of patients was significantly poorer in patients with low ALI than in patients with high ALI. **(E)** The Kaplan-Meier curve showed the cancer-specific survival of patients was significantly poorer in stage I+II patients with low ALI than patients with high ALI. **(F)** The Kaplan-Meier curve showed the survival of patients was not significantly different in stage III+IV patients with low ALI than patients with high ALI. ALI, advanced lung cancer inflammation index; HCC, hepatocellular carcinoma; TNM, tumor-node-metastasis.

Supplementary Figure 2 Forrest plot of the univariate and multivariate Cox regression analysis in HCC. **(A)** Forrest plot of the univariate and multivariate Cox regression analysis of overall survival; **(B)** Forrest plot of the univariate and multivariate Cox regression analysis of cancer-specific survival. AFP, alpha-fetoprotein; ALI, advanced lung cancer inflammation index; HCC, hepatocellular carcinoma; MVI, microvascular invasion; TNM, tumor-node-metastasis.

Supplementary Figure 3 Forrest plot of the subgroup survival using univariate Cox regression analysis in HCC. AFP, alpha-fetoprotein; ALI, advanced lung cancer inflammation index; CSS, cancer-specific survival; HCC, hepatocellular carcinoma; MVI, microvascular invasion; OS, overall survival; TNM, tumor-node-metastasis.
