# Supplementary figures and images for: Advanced lung cancer inflammation index predicts overall survival of hepatocellular carcinoma after hepatectomy
Source: Front Oncol. 2024 Feb 8;14:1294253. doi: 10.3389/fonc.2024.1294253 (PMC10882069; doi:10.3389/fonc.2024.1294253)

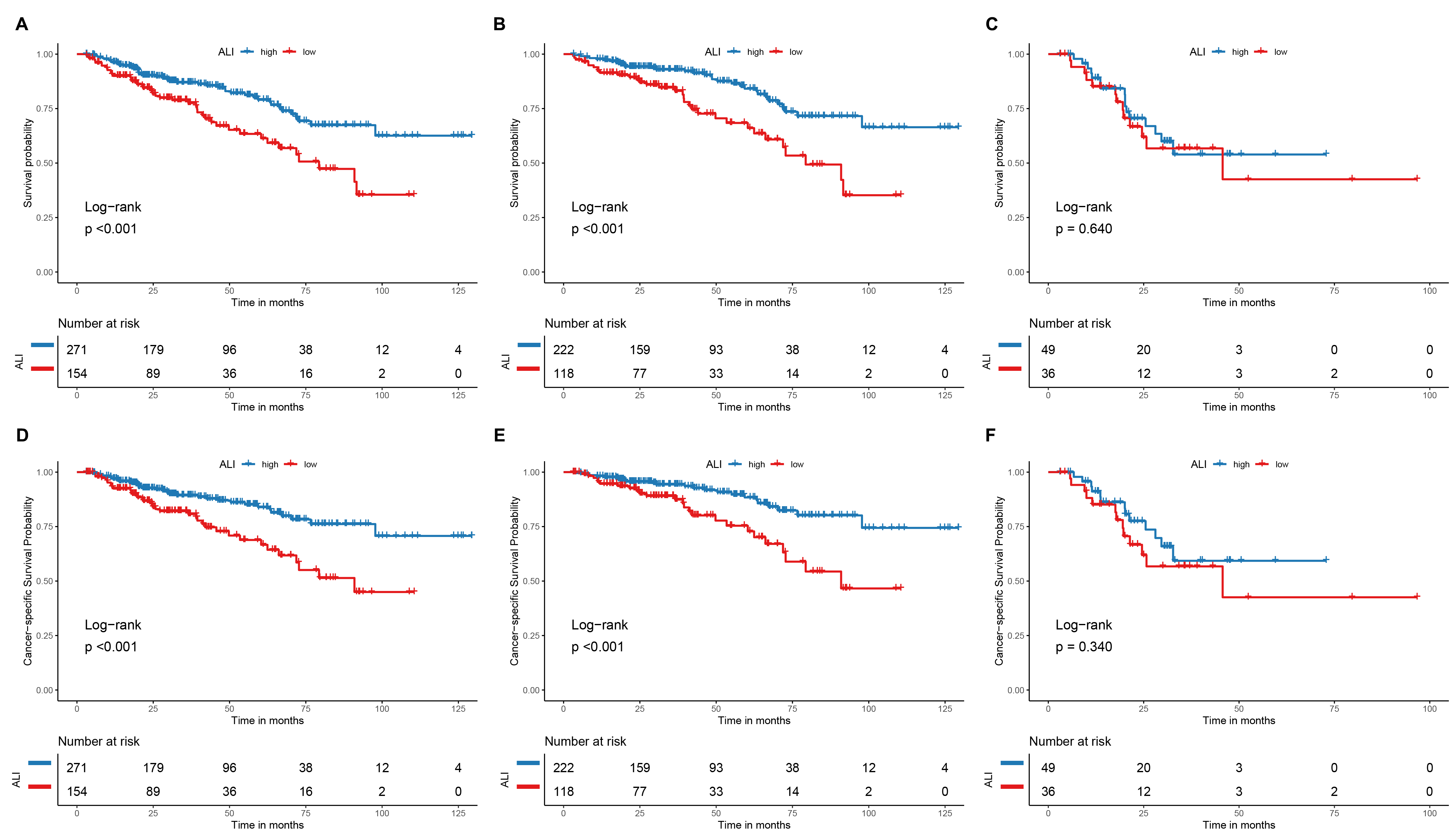

Supplement: Supplementary Figure 1 — The Kaplan-Meier curve for the ALI in HCC. (A) The Kaplan-Meier curve showed the survival of patients was significantly poorer in patients with low ALI than in patients with high ALI. (B) The Kaplan-Meier curve showed the survival of patients was significantly poorer in stage I+II patients with low ALI than patients with high ALI. (C) The Kaplan-Meier curve showed the survival of patients was not significantly different in stage III+IV patients with low ALI than patients with high ALI. (D) The Kaplan-Meier curve showed the cancer-specific survival of patients was significantly poorer in patients with low ALI than in patients with high ALI. (E) The Kaplan-Meier curve showed the cancer-specific survival of patients was significantly poorer in stage I+II patients with low ALI than patients with high ALI. (F) The Kaplan-Meier curve showed the survival of patients was not significantly different in stage III+IV patients with low ALI than patients with high ALI. ALI, advanced lung cancer inflammation index; HCC, hepatocellular carcinoma; TNM, tumor-node-metastasis. [file Image_1.tif]

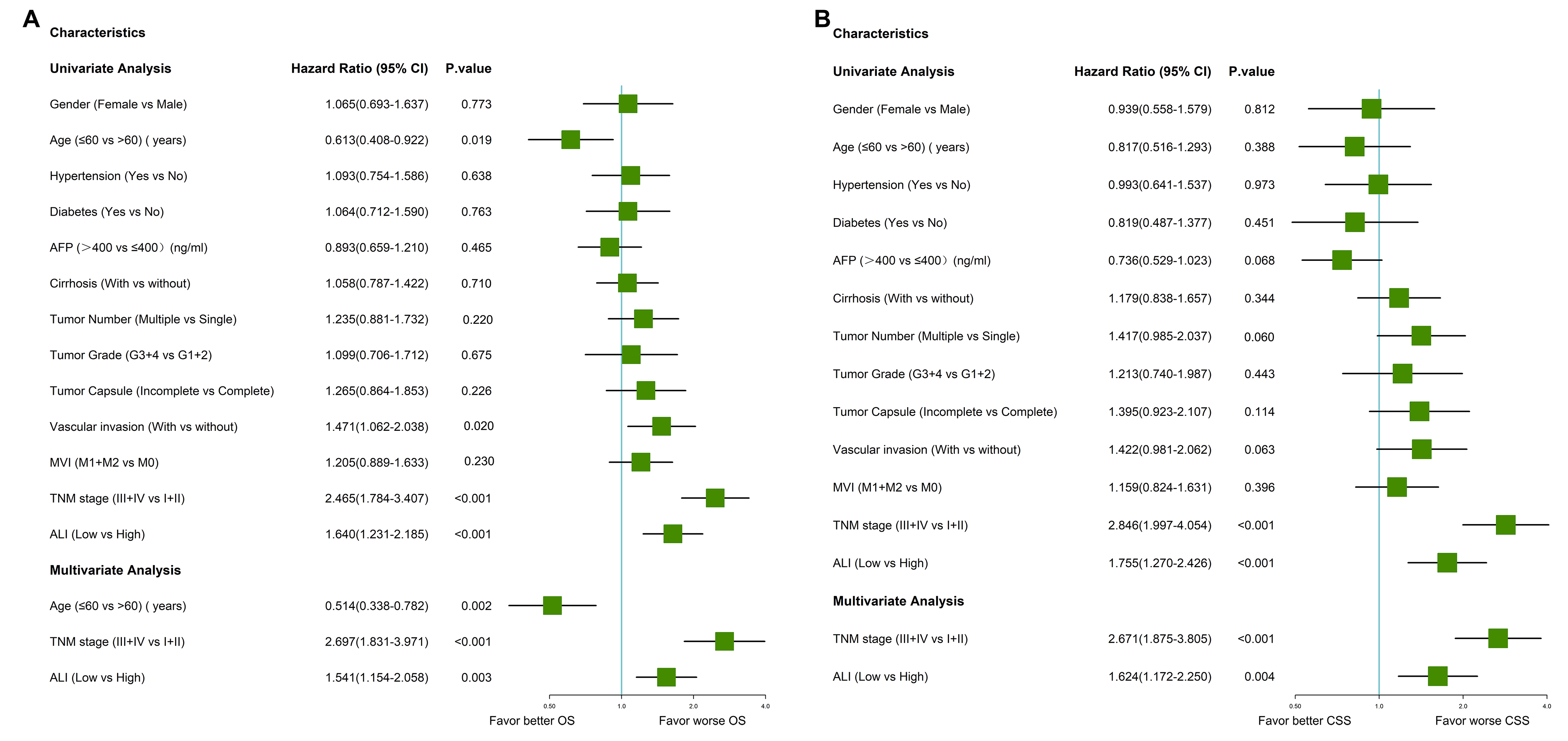

Supplement: Supplementary Figure 2 — Forrest plot of the univariate and multivariate Cox regression analysis in HCC. (A) Forrest plot of the univariate and multivariate Cox regression analysis of overall survival; (B) Forrest plot of the univariate and multivariate Cox regression analysis of cancer-specific survival. AFP, alpha-fetoprotein; ALI, advanced lung cancer inflammation index; HCC, hepatocellular carcinoma; MVI, microvascular invasion; TNM, tumor-node-metastasis. [file Image_2.tif]

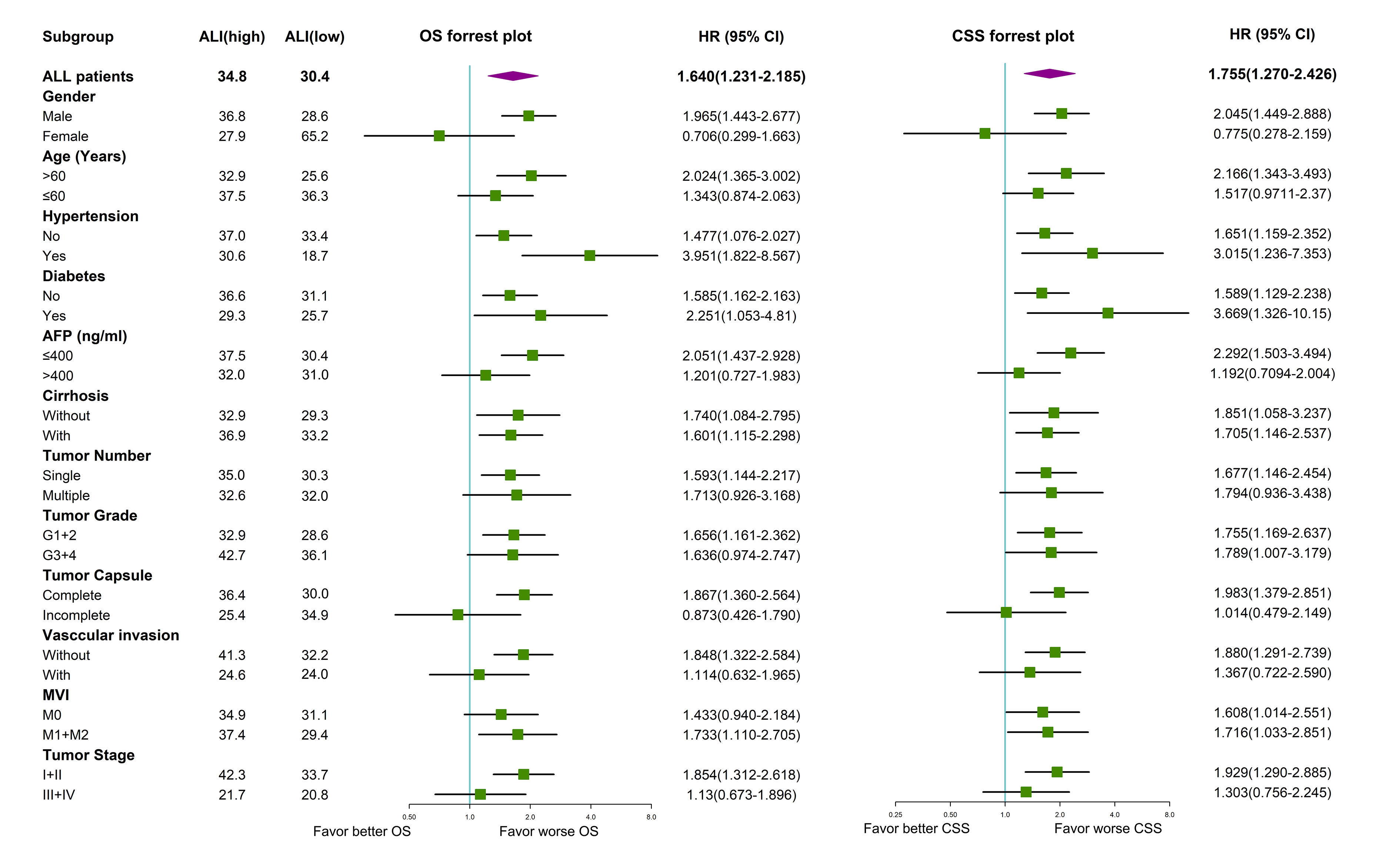

Supplement: Supplementary Figure 3 — Forrest plot of the subgroup survival using univariate Cox regression analysis in HCC. AFP, alpha-fetoprotein; ALI, advanced lung cancer inflammation index; CSS, cancer-specific survival; HCC, hepatocellular carcinoma; MVI, microvascular invasion; OS, overall survival; TNM, tumor-node-metastasis. [file Image_3.tif]
